# Efficient production of recombinant IgG by the GLUT5 co-expression system

**DOI:** 10.1186/1753-6561-5-S8-P50

**Published:** 2011-11-22

**Authors:** Yuichi Inoue, Aiko Inoue, Hiroharu Kawahara

**Affiliations:** 1 The Cell Engineering Center, Kitakyushu National College of Technology, Kitakyushu, 802-0985, Japan; 2 KYURIN CORPORATION, Kitakyushu, 806-0046, Japan

## 

A fructose containing cell culture medium has the advantage of low lactate production and a small pH change, leading to cell and product stability. But, not all cell lines grow well in the medium, and the fructose transporter, GLUT5, is related to it [1]. Thus, we developed an efficient production system of recombinant proteins by metabolic control and co-expression with GLUT5 in a fructose-based medium [2]. In this report, the availability of the GLUT5 co-expression system was indicated in CHO-K1 and the human cell line, SC-01MFP [3].

As a model, an IgG and GLUT5 co-expression vector was constructed and transfected into cells. When the transfected CHO-K1 and SC-01MFP cells were cultured in the fructose-based medium, both IgG productions were increased up to about two-fold of that cultured in the glucose-based medium (Table 1). Our study may be useful for efficient production of recombinant proteins using the fructose-based cell culture. In particular, the production in SC-01MFP cells is valuable for functional analysis of recombinant proteins with a human glycosylation profile.

**Table 1:** Proliferation and IgG production in the fructose-based medium.

Cell line	Relative cell proliferation	Relative IgG production
CHO-GLUT5/IgG	1.02 ± 0.19	1.77 ± 0.59
SC-01-GLUT5/IgG	0.86 ± 0.01	1.84 ± 0.04

Cells (1 x 10^5^ cells/ml) were cultured in the glucose- and fructose-based media. After 3 days, cell proliferation and recombinant IgG production were compared between two media. Each value in the glucose-based culture is estimated as 1.00. Data represent relative values of means ± SD (n = 3).
